# Effects of pulverized oyster mushroom *(Pleurotus ostreatus)* on diarrhea incidence, growth performance, immunity, and microbial composition in piglets

**DOI:** 10.1002/jsfa.9582

**Published:** 2019-03-04

**Authors:** Seidu Adams, Dongsheng Che, Jiang Hailong, Bao Zhao, Han Rui, Kofi Danquah, Guixin Qin

**Affiliations:** ^1^ Department of Animal Nutrition and Feed Science, College of Animal Science and Technology Jilin Agricultural University Changchun P.R. China; ^2^ School of Allied Health Sciences, Department of Nutritional Sciences University for Development Studies Tamale Ghana

**Keywords:** oyster mushroom, immunity, production performance, piglets, microbial composition

## Abstract

**BACKGROUND:**

*Pleurotus ostreatus* mushroom (POM) is an edible mushroom with rich nutritional components and vital pharmacological properties. The present study comprised 100 cross‐bred piglets, weaned at 28 days old, who were randomly assigned to four POM diets with five replicates per diet and five piglets per pen.

**RESULTS:**

POM supplementation (*P* < 0.05) decreased the incidence of diarrhea, and also increased the average daily feed intake and average daily gain of pigs. Fecal acetate, butyrate and propionate increased with the addition of POM. Interleukin‐2, immunoglobulin G, immunoglobulin M, tumor necrosis factor‐α and immunoglobulin A increased (*P* < 0.05) with the addition of POM. The 16S rDNA sequencing results showed that the *Bacteroidetes* and *Firmicutes* were the dominant microbial strains in the fecal samples, irrespective of POM supplementation. Shannon diversity, whole tree phylogenetic diversity, observed species and Chao1 analysis exhibited significant variation in species richness across the treatments. Principal coordinates analysis showed a significant (*P* < 0.1) increase in the microbial communities amongst all of the treatment groups.

**CONCLUSION:**

The results of the present study suggest that the supplementation of POM in the diet of piglets might increase feed consumption, gut microbial composition and diversity, as well as short‐chain fatty acids synthesis, consequently preventing the occurrence of diarrhea and increasing the growth of piglets. © 2019 The Authors. *Journal of The Science of Food and Agriculture* published by John Wiley & Sons Ltd on behalf of Society of Chemical Industry.

## INTRODUCTION

The use of antibiotics in non‐ruminant feed as a growth enhancer is valuable with respect to promoting growth parameters and disease control. A total reliance on antibiotics has led to the growth of resistant pathogens, with their accumulation in animal products resulting in a ban of the use of antibiotics as a growth promoter in animal feed.^1^ However, improving production and maintaining healthy livestock is important for commercial animal production. Early weaning is a usual husbandry practice in most commercial pig farms. This practice has a dramatic effect on the early life of piglets. Piglets during this period have an immature digestive system. Adapting to a drastic change from digestible watery milk to a less digestible solid feed appears to be challenging.^2^, ^3^ Furthermore, early weaning piglets are limited from the immunological and additional protection derived from the maternal milk, leading to an increase in their susceptibility to diseases and infection by enteric bacteria such as *Escherichia coli* and *Salmonella*.^4^, ^5^, ^6^ As a result of immature immune systems, insufficient nutrient utilization and other antigenic effects, their intestinal immunity is easily affected.^7^

Conversely, recent years have witnessed the use of different feed additives, such as plant extracts, organic acids, probiotics and prebiotics, in animal nutrition as a replacement for supplementation with antibiotics. Mushrooms are a macro‐fungi containing biologically active compounds and nutrients, such as sugars, amino acids,^8^ high fibres and protein, and they also have a low‐fat content.^9^ These biologically active compounds are either antioxidants or polysaccharides, which protect body cells against free radicals.^10^

Generally, oyster mushrooms are used as a vital component in traditional Chinese medicines and as a food for most people in Asia.^11^ These mushrooms contain a high amount of carbohydrates, minerals, proteins and vitamins, and have a low‐fat content.^12^ The key pharmacological properties of these mushrooms, including anti‐tumor, anti‐inflammatory, anti‐genotoxic, antioxidant, antihypertensive, antiplatelet aggregation agent, antimicrobial, antihyperglycemic, antiviral, hypocholesterolemic and immunomodulation properties of various *Pleurotus* sp., have been reported in rats, humans and other monogastrics.^13^ We hypothesize that the addition of the exact quantity of *Pleurotus ostreatus* mushroom (POM) to the diet of early weaning piglets may increase their growth performance and immunity, as well as prevent harsh weaning conditions. Therefore, the present study aimed to evaluate the effects of POM supplementation on the growth performance, immunity, nutrient digestibility, fecal pH, short‐chain fatty acids (SCFAs), microflora composition and the incidence of diarrhea in early weaning piglets.

## MATERIALS AND METHODS

### Animals

The present study was conducted in accordance with the research ethics and animal welfare and provincial pig rules and regulations of Jilin Agricultural University (Changchun, China). The study used 100 crossbred (Duroc × Large white × Landrace) piglets weaned at 28 days of age with a mean body weight of 8.92 ± 1.18 kg.

### Mushroom preparation and analysis

The POM were purchased from a local market (Jilin, Changchun, China). POM were dried overnight at 60 °C, pulverized through a 5 mm sieve and then stored in a closed vessel at room temperature for biochemical analysis before being incorporated into the experimental diets. The biochemical composition of the dry *P. ostreatus* samples was estimated using gas chromatography–mass spectrometry (GC/MS) method as described by Eman Mostafa^14^ (Table 1).

**Table 1:** Proximate analysis and GC/MS analysis of bioactive compounds in *Pleurotus ostreatus* (dry matter basis)

Component	Relative proportion
Moisture (g kg^−1^)	894.00
Total protein (N × 6.25) (g kg^−1^)	30.50
Crude fat (g kg^−1^)	3.00
Crude fiber (g kg^−1^)	18.00
Ash (g kg^−1^)	8.92
Dry matter (carbohydrates)	5.42
Starch (g kg^−1^)	7.00
Nitrogen free extract (g kg^−1^)	4.00
Total phenolic content (mg GAE g^−1^)	2.18
Terpenoids (mg GAE g^−1^)	1.05–9.99
Fatty acid (mg GAE g^−1^)	0.52–20.51
Alcohols (mg GAE g^−1^)	1.71
Heterocyclic (mg GAE g^−1^)	1.06

GAE, gallic acid equivalent.

### Experimental protocol

The piglets were divided into four experimental groups, with each group comprising five replicates with five piglets per replication pen, arranged in a complete randomized block design. The animals were housed in pens (1.8 × 1.2 m) with a plastic slatted floor. The pens were cleaned on a daily basis to prevent disease outbreak. The pre‐feeding exposure of piglets to the experimental diets lasted for 1 week prior to beginning the feeding trial. Four experimental diets containing 0.00, 5.00, 10.00 and 15.00 g kg^−1^ POM were formulated in accordance with the national nutrient requirements of swine.^15^ The diets contained soybean meal and cracked corn as the major sources of protein and energy, respectively (Table 2). The piglets were fed twice a day at 5:30 am and 5:30 pm with access to clean drinking water available *ad libitum*. Feed intake and orts were recorded daily in the morning on a pen basis.

**Table 2:** Ingredient levels and chemical composition of experimental diets (g kg^−1^ as fed) fed to determine the effects of various levels of dietary supplementation of *Pleurotus ostreatus* mushroom on performance, immunity and fecal microbial composition and functions in weaning pigs

	Dietary POM level (g kg^−1^)			
	0.00	5.00	10.00	15.00
Ingredient level				
Cracked corn	61.53	59.5	56.79	54.30
Soybean meal	20.90	18.63	14.40	14.63
Fish meal	3.66	3.66	3.46	2.30
Whey powder	3.41	3.41	4.35	4.35
Fermented soybean meal	6.50	8.20	12.00	12.92
POM^a^	0.00	5.00	10.00	15.00
Premix^b^	4.00	4.00	4.00	4.00
Chemical composition				
ME (MJ kg ^−1^)	10.39	10.27	10.36	10.13
Crude protein	19.91	19.93	19.86	19.89
Methionine	0.36	0.35	0.34	0.32
Lysine	1.24	1.22	1.21	1.18
Calcium	0.82	0.81	0.81	0.80
Available Phosphorus	0.41	0.39	0.36	0.38

a POM; different dietary concentrations of *P. ostreatus* mushroom prepared by drying overnight at 60 °C and pulverized to pass through a 5 mm sieve. 0.00 g kg^−1^ POM, 5.00 g kg^−1^ POM, 10.00 g kg^−1^ POM and 15.00 g kg^−1^ POM.

b Premix provides the following per kg: Vitamin A 130‐396 KIU, Kilo‐vitamin D 30–124 KIU, Vitamin E 400mg, Vitamin K_2_ 40–150mg, vitamin B_2_ 75–1500 mg, 4500–1500 mg, Iron 1500‐3700 ppm, Magnesium 400–3700 ppm, moisture 9%, sodium 6–14%, total Phosphorus 2.0 %, lysine 1.3 %, Calcium 10–20 %, Phytase 12500 U.

### Diarrhea incidence

The piglets were monitored daily for signs of diarrhea; those with watery feces were classified either as pasty or fluid and recorded as a diarrhea case.^16^ The diarrhea occurrence (g) was estimated as described by Hu *et al.* 17

### Growth performance and nutrient digestibility

The piglets were weighed early in the morning prior to feeding on the first and last days of the feeding trials. The final body weight minus the initial body weight and divided by the number of days of the experiment was used to obtain the average daily gain (ADG). The average daily feed intake (ADFI) was determined by calculating the total feed consumed per day. The feed intake was divided by the body weight gain of piglets to obtain the feed conversion ratio (FCR). On the last day of the experiment, 100 g of fresh fecal samples were collected directly from the rectum of pigs through rectal massage. The fecal samples were homogenized and stored at −20 °C until further laboratory analysis. The samples were analyzed for crude protein, crude fibre, gross energy and ether extract digestibility in accordance with the procedures described by Hassanat *et al.* 18

### Immunological parameters, fecal pH and SCFAs

At the end of the experiment, blood samples were collected into 10‐mL tubes via jugular vein puncture and centrifuged at 3000 × *g* and 4 °C for 10 min to recover the serum using the Eppendorf® Centrifuge 5810/5810R analyzer (MilliporeSigma, Burlington, MA, USA) and blood sera were collected to analyze for serum cytokines [tumour necrosis factor (TNF)‐α, interleukin (IL)‐2] and immunoglobulins (Ig) (IgA, IgG and IgM) using porcine enzyme‐linked immunoabsorbent assay (ELISA) kits: TNF‐α ELISA Kit (ab100756), IL‐2 ELISA Kit (ABIN365284), Pig IgA ELISA Kit (ab190536), Pig IgG ELISA Kit (ABIN431274) and Pig IgA ELISA Kit (ab190536) in accordance with the manufacturer's instructions (Abcam, Cambridge, MA, USA). Fecal pH was measured using a portable pH meter. The three main SCFAs (acetate, butyrate and propionate) were analyzed in accordance with the methods described by Freire *et al.* 19 Another 10 g of fresh fecal samples was collected on a pen basis, stored on an ice cooler and then transported to the laboratory for fecal microbial analysis.

### Total DNA extraction

Extraction of the total DNA was performed as described by Corrigan *et al.* 20 using a QIAamp DNA Mini Stool Kit (Qiagen, Germantown, MD, USA). Briefly, a sample of fecal content was pulverized to a fine powder using a mortar and pestle, combined with proteinase, transferred to a centrifuge tube and then incubated for 40 min at 50–55 °C. The samples were centrifuged at 10000 × *g* for 10 min and the supernatant was removed and combined with a preheated 2% agarose mixture, followed by washing in a 10% volume of Tris‐ethylenediaminetetraacetic acid buffer. The genomic DNA was quality checked and quantified using a Nanodrop 2000C spectrophotometer (ThermoFisher, Waltham, MA, USA).

### 16S rDNA amplification and sequencing of the V3–V4 region

The extracted DNA from the fecal samples was amplified using two sets of bacterial: 341F (5'‐CCTACACGACGCTCTTCCGATCTN‐3') and 805R (5'‐GACTGGAGTTCCTTGGCACCCGAGAATTCCA‐3'), in accordance with the methods described by Logares *et al.* 21 The hypervariable V3–V4 regions of the bacterial 16S rDNA were amplified using a polymerase chain reaction (PCR) at 95 °C for 5 min denaturation, followed by 25 cycles at 95 °C for 30 s, 55 °C for 30 s and 72 °C for 30 s, with extension at 72 °C for 5 min. The PCR was performed in a mixture containing reagents and 50 ng of template DNA, in accordance with the procedure described by Drumo *et al.* 22 The amplicons were extracted using 2% agarose gel electrophoresis and the DNA was recovered using the agarose recovery kits. The PCR amplified product size was selected using a Qubit 2.0 DNA assay kit (ThermoFisher) and pooled in equimolar concentration and paired‐end sequenced on the Illumina platform (Hiseq or Miseq) (Illumina, Inc., San Diego, CA, USA). The paired‐end reads were demultiplexed and quality filtered using the following control standard criteria: (i) the 250‐bp reads were removed from any site receiving an average quality score of less than 20; (ii) exact barcode matching, two nucleotide mismatch and reads with ambiguous characters were removed; and (iii) reads that ranged between 220 and 500 nucleotides were assembled according to their overlap sequence.

### Operational taxonomic units (OTUs) picking and phylogenetic diversity analysis

The OTUs were clustered with 97% similarity cut‐off using USEARCH (https://www.drive5.com/usearch) and chimeric sequences, subsequently filtered out to obtain OTUs for species classification.^23^ Each sequence was randomly analyzed to avoid biasness as a result of sample size differences. The dudi.pca function was used to perform the principal component analysis (PCoA) to determine the status of the treatments. The most abundant sequence in each OTU was selected as the representative sequence, then aligned against the core set of green genes in the 16S database (http://greengenes.lbl.gov). After classification, the OTU index table was obtained according to the number of sequence in each OTU.^24^

### Formation of functional profiles

The sequence number in each OTU was arranged in heat maps using the R language g‐plot package (R Foundation for Statistical Computing, Vienna, Austria) in which the color gradient reflects the abundance of the species in different sample clusters. The sample alpha diversity index was determined using QIME, version 1.7 (http://qiime.org) to create corresponding dilution curves based on the relative proportion of the OTUs to the 16S rDNA sequence.^25^ The beta diversity index was used to analyze sample species complexity. Linear discrimination analysis was also used to estimate the treatment effects on the species abundances.

### Statistical analysis

The GLM procedures of SPSS, version 20.0 (IBM Corp., Armonk, NY, USA) were used to determine the treatment effects using one‐way analysis of variance. Polynomial contrasts (linear, quadratic and cubic) were used to test the effects of different POM levels on the various parameters measured.

## RESULTS

### Effects of dietary POM levels on diarrhea incidence in weaning pigs

The diarrhea incidence decreased when 5.00, 10.00 and 15 g kg^−1^ POM diets were fed compared to the 0.00 g kg^−1^ POM diet (linear, quadratic and cubic effects, *P* < 0.05) (Table 3).

**Table 3:** Performance and diarrhea incidence of weaning pigs fed diets containing different levels of POM^a^

	Dietary POM levels (g kg^−1^)					Polynomial contrasts (*r* ^2^)^b^			
	0.00	5.00	10.00	15.00	SEM	Linear	Quadratic	Cubic	*P* value
ADFI (g)	497.11	411.27	497.34	553.85	4.73	0.0295	0.7942	1.0000	0.001
ADG (g)	285.64	191.71	289.57	469.21	15.44	0.3279	0.9842	1.0000	0.005
FCR	2.75	3.15	2.72	2.18	0.67	0.2607	0.9427	1.0000	0.005
DIARRHEA (g)	6.54	3.23	3.73	2.93	0.32	0.5138	0.8327	1.0000	< 0.001

a POM; different dietary concentrations of *P. ostreatus* mushroom prepared by drying overnight at 60 °C and pulverized to pass through a 5 mm sieve.

b Polynomial contrasts (*r* ^2^).

ADFI, average daily feed intake; ADG, average daily gain; FCR, feed conversion ratio; DIARRHEA, diarrhea incidence in pigs.

### Effects of dietary POM levels on the growth performance of weaning pigs

The feed intake increased when 10.00 and 15.00 g kg^−1^ POM diets were fed compared to the 5.00 and 0.00 g kg^−1^ POM diets (quadratic and cubic effects, *P* < 0.05; linear effect, *P* > 0.05) (Table 3). When higher levels (10.00 and 15.00 g kg^−1^) of POM were fed, the growth rate increased compared to the growth rate obtained when 5.00 and 0.00 g kg^−1^ POM diets were provided (quadratic and cubic effects, *P* < 0.05; linear effect, *P* > 0.05). The feed conversion was significantly (quadratic and cubic effects, *P* < 0.05; linear effect, *P* > 0.05) improved with the addition of the POM. The highest feed efficiency was observed in the 15.00 g kg^−1^ POM group.

### Effects of POM on the nutrient digestibility of weaning pigs

The crude protein content of the 5.00 and 15.00 g kg^−1^ POM was intermediate compared to the crude protein content obtained when 0.00 and 10.00 g kg^−1^ POM diets were fed (Table 4, cubic effect, *P* < 0.05; linear and quadratic effects, *P* > 0.05). The crude fibre levels increased at a higher dietary POM level (10.00 g kg^−1^), reaching a plateau and then declining when the POM level was 15.00 g kg^−1^ (quadratic and cubic effects, *P* < 0.05; linear effect, *P* > 0.05). The ether extract increased when 5.00, 10.00 and 15.00 g kg^−1^ POM diets were fed compared to the 0.00 g kg^−1^ POM diet (linear, quadratic and cubic effects, *P* < 0.05). The gross energy increased at higher dietary POM level (15.00 g kg^−1^) and then reached a plateau (linear, quadratic and cubic effects, *P* < 0.05).

**Table 4:** Nutrient digestibility (g kg^−1^ as fed), pH and concentration of acetate (mmol L^−1^), butyrate (mmol L^−1^), propionate (mmol L^−1^) and total SCFA (mmol L^−1^) of weaning pigs fed diets containing different levels of POM^a^

	Dietary POM levels (g kg^−1^)					Polynomial contrasts (*r* ^2^)^b^			
	0.00	5.00	10.00	15.00	SEM	Linear	Quadratic	Cubic	*P* value
Crude protein	70.75	76.12	86.22	75.14	1.20	0.2098	0.3331	0.9279	0.002
Crude fiber	60.02	73.89	74.59	66.90	2.44	−0.161	0.9914	1.0000	0.003
Ether extract	69.06	74.99	74.41	88.45	1.43	0.8039	0.8851	1.0000	0.002
Gross energy	65.69	76.42	74.18	86.94	1.41	0.8200	0.8204	1.0000	0.003
PH	5.55	5.64	5.66	5.61	0.08	0.2681	0.5213	0.9462	0.135
Acetate	35.95	45.37	49.83	42.96	2.75	0.1225	0.9788	1.0000	0.010
Propionate	11.17	25.12	24.30	22.98	2.20	0.2381	0.9165	1.0000	0.017
Butyrate	9.13	10.39	11.14	13.35	1.22	0.9525	0.9783	1.0000	0.010
Total SCFA	56.25	80.88	85.27	79.29	5.69	0.3444	0.9899	1.0000	0.015

a POM; different dietary concentrations of *P. ostreatus* mushroom prepared by drying overnight at 60 °C and pulverized to pass through a 5 mm sieve.

b Polynomial contrasts (*r* ^2^).

### Effects of dietary POM levels on fecal pH and SCFAs

The fecal pH level increased with the increase in dietary POM levels (quadratic and cubic effects, *P* > 0.05; linear effect, *P* > 0.05). The highest pH levels (5.64 and 5.66) occurred in the 5.00 and 10.00 g kg^−1^ POM treated groups. The acetate levels increased with the increase in dietary POM concentrations (quadratic and cubic effects, *P* < 0.05; linear effect, *P* > 0.05). The highest acetate levels (45.37 and 49.83 mmol L^−1^) occurred in the 5.00 and 10.00 g kg^−1^ POM treated groups. The propionate level increased with the increase in dietary POM levels (quadratic and cubic effects, *P* < 0.05; linear effect, *P* > 0.05). The highest propionate levels (24.30 and 25.12 mmol L^−1^) occurred in the 5.00 and 10.00 g kg^−1^ POM treated groups. The butyrate levels increased with the increase in dietary POM levels (linear, quadratic and cubic effects, *P* < 0.05). The highest butyrate levels (11.14 and 13.35 mmol L^−1^) occurred in the 10.00 and 15.00 g kg^−1^ POM treated groups. The total SCFAs increased with the increase in dietary POM levels (quadratic and cubic effects, *P* < 0.05; linear effect, *P* > 0.05). The highest total SCFAs levels (80.88 and 85.27 mmol L^−1^) occurred in the 5.00 and 10.00 g kg^−1^ POM treated groups (Table 4).

### Effects of dietary POM levels on serum cytokines and immunoglobulins

The IL‐2 concentration first increased with the increase in dietary POM levels (quadratic and cubic effects, *P* < 0.05; linear effect, *P* > 0.05), reaching a plateau at 10.00 g kg^−1^ and then decreasing at higher dietary POM levels (15.00 g kg^−1^). The TNF‐α concentration increased with the increase in dietary POM levels (quadratic and cubic effects, *P* < 0.05; linear effect, *P* > 0.05). The highest TNF‐α levels (525.82 and 556.46 µg mL^−1^) occurred in the 5.00 and 15.00 g kg^−1^ POM treated groups. At higher POM levels (10.00 and 15.00 g kg^−1^), the IgA concentrations were intermediate to the IgA concentrations at 0.00 and 5.00 g kg^−1^ POM (quadratic and cubic effects, *P* < 0.05; linear effect, *P* > 0.05). The IgG concentration increased when 5.00 and 10.00 g kg^−1^ POM diets were fed compared to the IgG concentration when 0.00 and 15.00 g kg^−1^ POM diets were fed (quadratic and cubic effects, *P* < 0.05; linear effect, *P* > 0.05). The IgM concentrations increased with the addition of dietary POM levels (linear, quadratic and cubic effects, *P* < 0.05). The highest IgM concentrations (21.50 and 21.40 µg mL^−1^) occurred in the 10.00 and 15.00 g kg^−1^ POM treated groups, respectively (Table 5).

**Table 5:** Concentrations of IL‐2 (µg mL^−1^), TNF‐α (µg mL^−1^), IgA (µg mL^−1^), IgG (µg mL^−1^) and IgM (µg mL^−1^) of weaning pigs fed diets containing different levels of POM^a^

	Dietary POM levels (g kg^−1^)					Polynomial contrasts (*r* ^2^)^b^			
	0.00	5.00	10.00	15.00	SEM	Linear	Quadratic	Cubic	*P* value
IL‐2	632.03	846.10	1184.48	880.20	120.73	0.3011	0.8005	1.0000	0.030
TNF‐α	267.97	556.46	470.06	525.82	60.62	0.2676	0.7215	1.0000	0.002
IgA	159.07	180.23	167.50	165.83	6.95	−0.375	0.5459	1.0000	0.026
IgG	8.66	9.55	9.61	9.39	2.37	0.2086	0.9724	1.0000	0.034
IgM	13.83	14.23	21.50	21.40	4.04	0.8061	0.8065	1.0000	0.013

IL‐2, interleukin‐2; TNF‐α, tumor necrosis factor‐α; IgA, immunoglobulin A; IgG, immunoglobulin G; IgM, immunoglobulin M.

a POM; different dietary concentrations of *Pleurotus ostreatus* mushroom prepared by drying overnight at 60 °C and pulverized to pass through a 5 mm sieve.

b Polynomial contrasts (*r* ^2^).

### Effects of dietary POM levels on the fecal microbial composition

**Figure 1:**
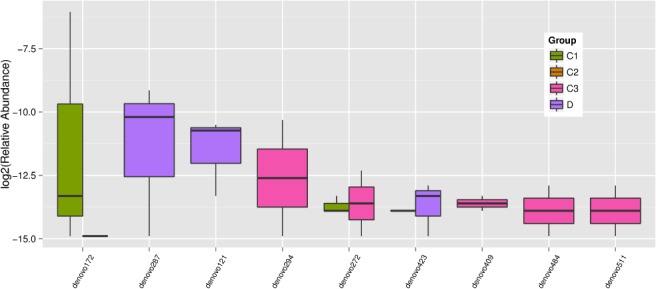
Showing the significant difference (*P* < 0.05) between the different treatment groups at the OTU reads. The colours represent the groups and the OTUs represent each organism.

**Figure 2:**
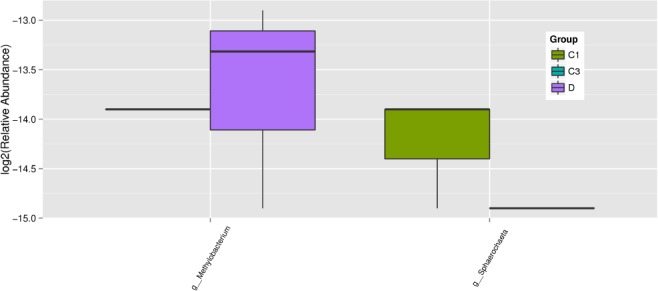
Showing the significant difference in relative abundance (*P* < 0.1) between the treatment groups and the control at the genus level. At the genus level, different colours represent the treatments. The relative abundance of g__*Methylobacterium* was significantly higher in the 0.00 g kg^−1^ POM compared to the treatment groups. The relative abundance of g_ *Sphaerochaeta* was significantly higher in the 5.00 g kg^−1^ POM treatment group compared to the other treatment.

**Figure 3:**
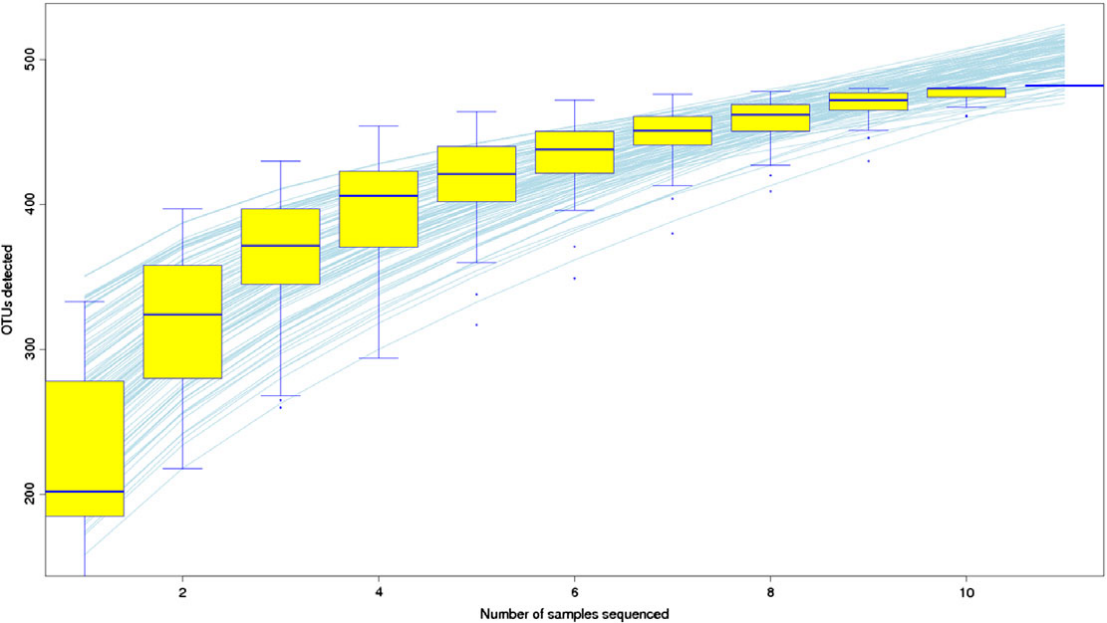
The rarefaction curves produced by the boxplots represent the number of sample sequences against the number of OTUs, and tended towards the saturation plateau.

**Figure 4:**
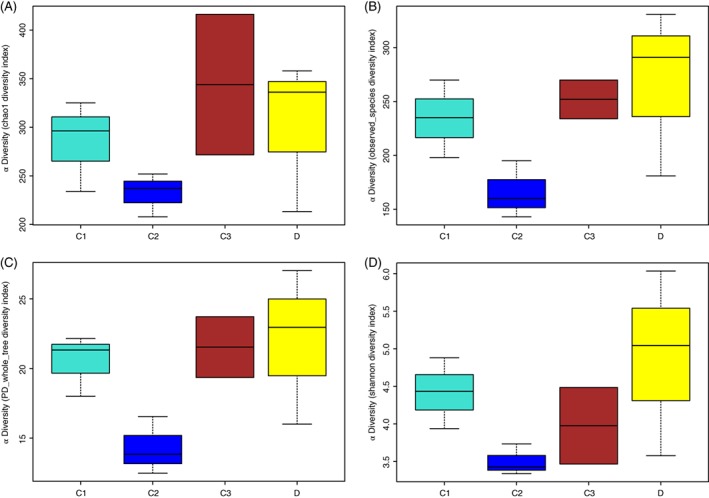
Showing the alpha diversity indices (wilcox.test function in R for both sets of samples and the kruskal.test function in R if more than two sets of samples were used). The observed species index (B) and the Chao index (A) reflect the species richness of the communities in the sample. The whole tree phylogenetic diversity index (C) reflects the species diversity of the communities affected by species richness and species evenness in the sample community. The Shannon index (D) reflects the difference in species preservation in the sample from evolutionary history.

The 16S rDNA gene pyrosequencing generated a total of 336 446 quality sequences, with an average of 232.45 per sample. The number of OTUs obtained was 482 based on the nucleotide sequence identity between the reads. The minimum and maximum number of OTUs obtained per sample was 143 and 333, respectively. Moreover, a significant difference was observed between the bacterial communities at the OTUs and the genus level amongst all of the treated groups (Figs 1 and 2). The samples provided sufficient OTU coverage to generate rarefaction curves with levels tending to plateau (Fig. 3). The Shannon diversity, whole tree phylogenetic diversity index, observed species and Chao1 index values for species richness were higher in the 15.00 g kg^−1^ POM treated group and the control group compared to the other treatment groups (Fig. 4). The rarefaction analysis indicated good sequencing results, which implies that the samples were sufficient for the estimation of the total bacterial population in the fecal microbiota of piglets.

**Figure 5:**
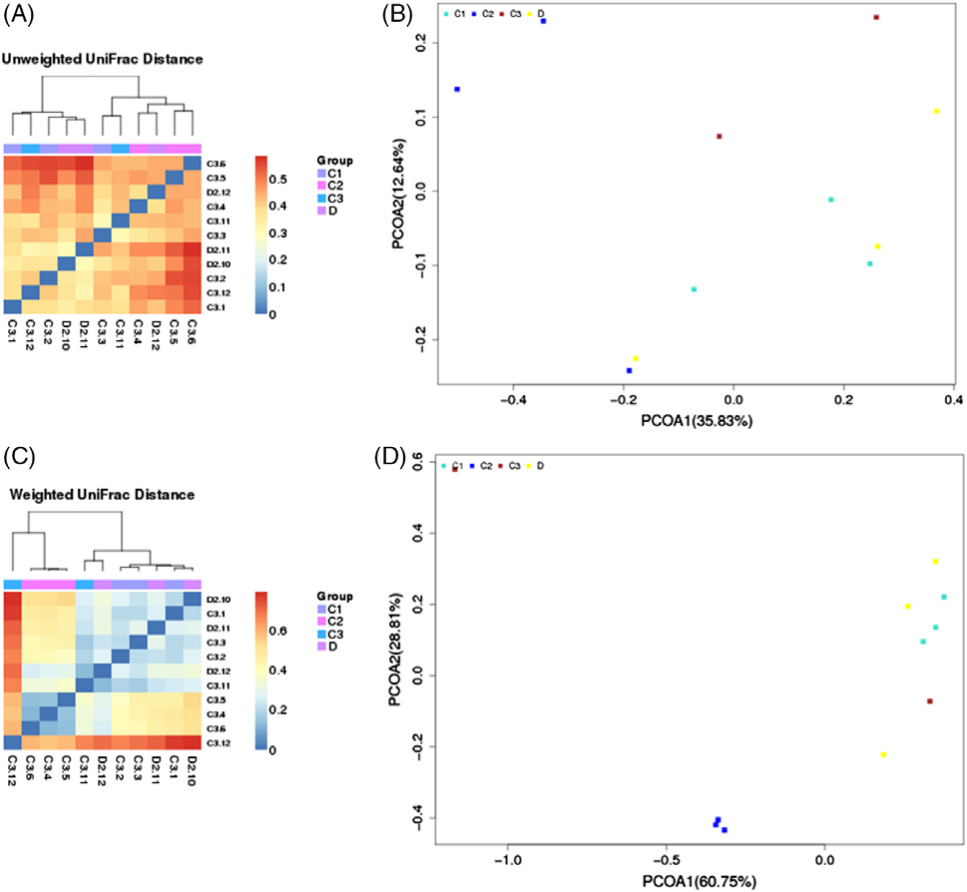
Showing the UniFrac distance distribution heat map with respect to clustering samples with similar beta diversity; the clustering UniFrac results reflects the similarity between samples. The UniFrac results are divided into two types: (A) (unweighted UniFrac) and (C) (weighted UniFrac). The weighted UniFrac considers the sequence abundance and the unweighted UniFrac does not consider the abundance. PCoA analysis [(B): Unweight_UniFrac] and [(D): Weight_UniFrac].

**Figure 6:**
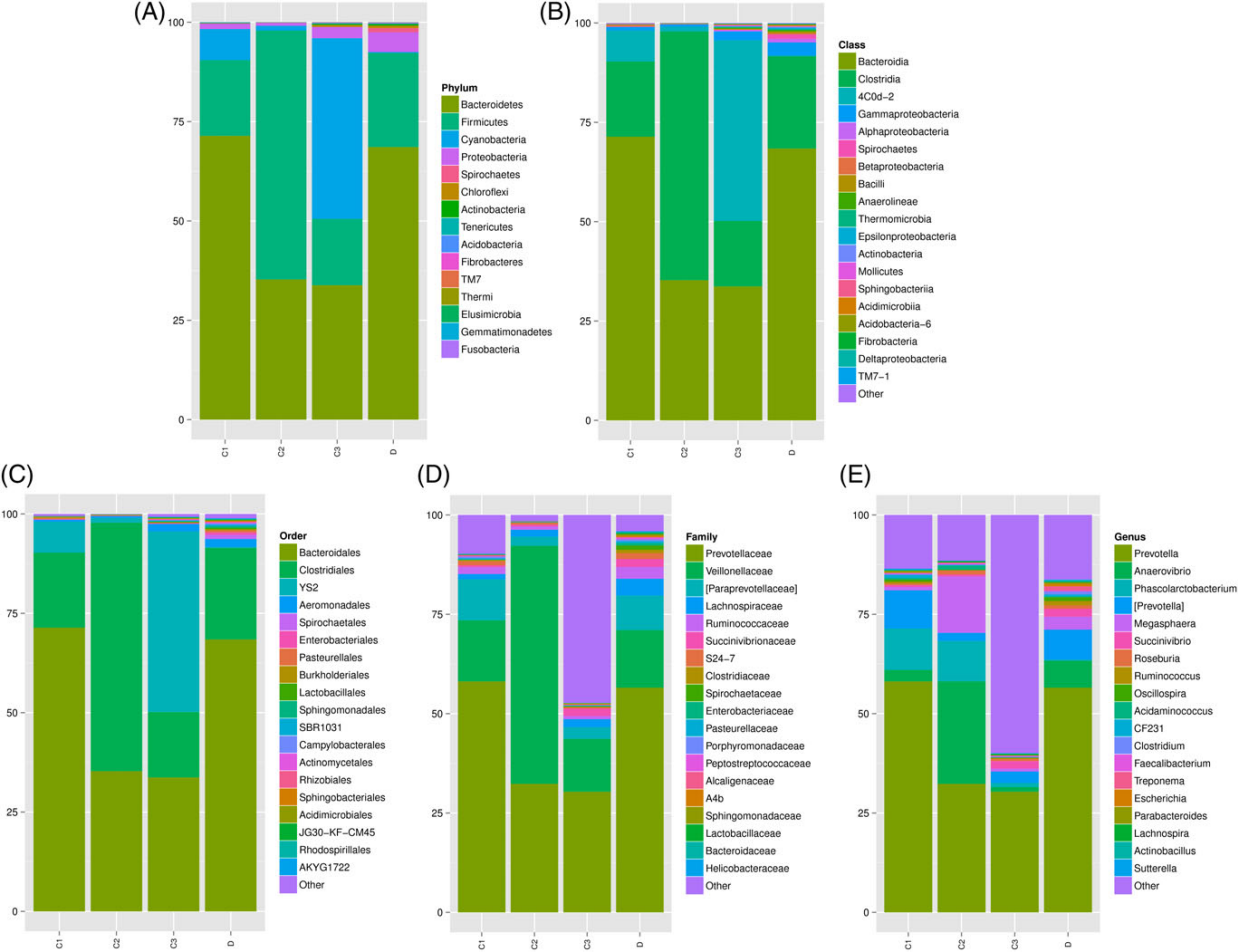
Microbial composition and diversity within the taxa. A; represents the microbial composition in the Phylum level, B; represents microbial composition in the Class level, C; the represents microbial composition in the Order level, D; the represents microbial composition in the Family level, and E; the represents microbial composition in the Genus level.

The results of the sample clustering showed the similarities and differences between the treated groups. The differences (*P* < 0.1) between the bacterial communities were observed in all of the treated groups (Fig. 5). At the phylum level, a total of 482 phyla was detected in all of the samples. The results showed that the *Bacteroidetes*, *Firmicutes*, *Cyanobacteria* and *Proteobacteria* were the dominant bacterial phyla, representing approximately 97% of the bacterial microbiota across the treated groups (Figs 4 and 6A). Comparatively, the *Spirochaetes*, *Chloroflexi* and *Actinobacteria* phyla were less predominant and their total proportion of all the treated samples was approximately 2% (Fig. 6A).

At the Class level, a total of 481 classes was observed across the treatments. The results showed that the *Bacteroidia*, *Clostridia*, 4Cod‐2 and *Gammaproteobacteria* were the dominant classes in the samples. The relative abundance of the *Bacteroidia* was significantly higher (*P* < 0.05) in the 5.00 g kg^−1^ POM group and the control group compared to the other treated groups, whereas *Clostridia* were higher (*P* < 0.05) in the 10.00 g kg^−1^ POM treated group. The relative abundance of 4Cod‐2 was significantly higher in the 15.00 g kg^−1^ POM treated group. The *Gammaproteobacteria*, *Alphaproteobacteria*, *Spirochaetes*, *Betaproteobacteria*, *Bacilli* and *Anaerolineae* were dominant in the control group compared to the other treated groups (Fig. 6B).

At the order level, a total of 437 orders was detected across the treatment groups. The results indicated that the *Bacteroidales*, *Clostridiales*, YS2, *Aeromonadales*, *Spirochaetales*, *Enterobacteriales*, *Pasteurellales* and *Burkholderiales* were dominant in all of the samples (Fig. 6C). The relative abundance of the *Bacteroidales* was significantly higher (*P* < 0.05) in the 5.00 g kg^−1^ POM treated group and the control group compared to the other treatments. The relative abundance of the *Clostridiales* was significantly higher (*P* < 0.05) in the 10.00 g kg^−1^ POM treated group compared to the other treatments. The relative abundance of YS2 was significantly higher (*P* < 0.05) in the 15.00 g kg^−1^ POM treated group compared to the other treatment groups. At the family level, a total of 379 families was detected across the samples. The results showed that the most dominant families in the samples were the *Prevotellaceae*, *Veillonellaceae*, [*Paraprevotellaceae*], *Lachnospiraceae*, *Ruminococcaceae*, *Succinivibrionaceae* and S24‐7 (Fig. 6D). The *Prevotellaceae* was relatively abundant (*P* < 0.05) in the 5.00 g kg^−1^ POM treated group and the control group compared to the other treatment groups. Furthermore, the *Veillonellaceae* was relatively abundant in the 10.00 g kg^−1^ POM treated group. The relative abundance of the uncharacterized families was significantly higher (*P* < 0.05) in the 15.00 g kg^−1^ POM compared to the other treatment groups. In addition, the [*Paraprevotellaceae*] was significantly higher in the 5.00 g kg^−1^ POM treated group and the control group compared to other treatment groups.

At the genus level, a total of 231 genera was detected across the treated samples. The *Prevotella*, *Anaerovibrio*, *Phascolarcprevotella*, [*Prevotella*], *Megasphaera*, *Succinivibrio*, *Roseburia*, *Ruminococcus* and *Oscillospira* (Fig. 6E) were the dominant genera in the samples. The results showed that the relative abundance of the main bacteria genera was significantly (*P* < 0.05) affected by the supplementation of POM. The relative abundance of the *Prevotella* was significantly higher (*P* < 0.05) in the 5.00 g kg^−1^ POM treated group and the control group. The relative abundance of the *Anaerovibrio* was significantly higher (*P* < 0.05) in the 10.00 g kg^−1^ POM group compared to the other treatment groups. The relative abundance of the uncharacterized genus was significantly higher (*P* < 0.05) in the 15.00 g kg^−1^ POM treated group. The [*Prevotella*], *Megasphaera*, *Succinivibrio*, *Roseburia* and *Ruminococcus* were dominant in the control group compared to the POM treated groups.

## DISCUSSION

### Effects of dietary POM levels on diarrhea incidence of weaning pigs

The incidence of diarrhea in weaning piglets is caused by an overpopulation of the coliform bacteria and the control of its spread is an effective means of reducing diarrhea incidence in piglets.^26^ The results of the present study showed that the supplementation of POM lowered (*P* < 0.05) diarrhea incidence in piglets. The results were in agreement with our previous observations,^27^, ^28^ in which dietary supplementation with *Astragalus membranaceus* (medicinal plant) fibre significantly decreased diarrhea incidence in piglets. Similarly, Huang *et al.* 29 observed a reduction in diarrhea incidence and the diarrhea index in piglets treated with complex *Lactobacilli* preparations compared to the control. In addition, Li *et al.* 26 observed a reduction in diarrhea incidence after supplementing *Atractylodes macrophylla* Koidz polysaccharides in the diet of early weaning piglets. The results of the present study imply that the bioactive compounds such as fibre, glucans, phenols and other active ingredients present in POM may effectively decrease the enteric bacteria (*Coliform, E. coli*, etc.) population and activities in the intestines of piglets, hence decreasing diarrhea incidence. However, the mechanism of action involved in this process is unclear.

### Effects of dietary POM levels on the growth performance of weaning pigs

Early weaning is a harsh condition in the early life of piglets. Piglets in this condition are exposed to several disorders, such as impaired intestinal metabolism, immune functions, growth performance and mortality.^26^ The present study revealed that the inclusion of POM in the diet of piglets increased the ADFI, resulting in an increased in the growth rate and consequently improved the feed to gain ratio of the entire studies. Mushrooms contain some bioactive compounds such as vitamins, minerals and other chemical compounds that may increase the feed consumption of piglets hence increase their body weight gain. The results of the present study were in agreement with the findings of Song *et al.* 30 who found that the ADFI and FCR significantly (*P* < 0.05) increased with the addition of fermented oyster mushroom by‐products in the diet of pigs. In addition, dietary supplementation with *A. membranaceus* fibre increased the growth performance of piglets.^27^, ^28^ Also, Li *et al.* 26 reported that dietary supplementation with *A. macrophylla* Koidz polysaccharides improved the ADG and FCR of weaning piglets. Moreover, there was an increased in the growth performance of broiler chicken after dietary supplementation with *Agaricus bisporus*,^31^ shiitake mushroom extract,^32^ *Lentinus edodes* and *Tremella fuciformis* 33 mushrooms. By contrast, Daneshmand *et al.* 34 observed a suppression in feed intake and body weight gain after supplementing 2 g kg^−1^ oyster mushroom (*P. ostreatus*) in the diet of male broiler birds. Daneshmand *et al.* 34 concluded that different pulverized mushrooms (*L. edodes* and *T. fuciformis*) had no significant effect on the body weight of birds. Therefore, the difference between the results of the present study and those previous studies might be a result of the difference in concentration and the breed of animals used.

### Effects of POM on the nutrient digestibility of weaning pigs

The digestibility of nutritional components is a major factor in feed formulation for early weaning piglets. During weaning and immediately after weaning, a piglet's digestive tract is not well developed and hence the digestion of most feed ingredient is compromised. Kiczorowska *et al.* 35 observed that the digestibility of most nutrients was influenced by the intestinal diameter and other physical factors such as the microbial biosphere. Mushrooms contain some biologically active compounds and nutrients, such as sugars, amino acids,^8^ high fibres, protein and low‐fat content.^9^ These nutritionally active compounds may have significant effects on the nutrient digestibility of animals, as well as humans. In the present study, the digestion of crude protein, ether extract, gross energy, and crude fibre increased (*P* < 0.05) in the POM treated groups compared to the control. The results of the present study were in agreement with the findings by Che *et al.* 27 who reported that the supplementation of low concentration (2.50%) of *A. membranaceus* fibre increased the digestibility of crude protein, crude fibre, gross energy and dry matter in early weaned piglets. By contrast to the present study, Che *et al.* 27 observed that the digestibility of crude protein, crude fibre, gross energy and dry matter decreased as the concentration of *A. membranaceus* fibre increased in the diet of piglets. Similarly, Jørgensen *et al.* 36 reported that the supplementation of high dietary fibre to the diet of pigs decreased the crude protein and gross energy digestibility. In addition, Dilger *et al.* 37 observed a decrease in the digestibility of gross energy in pigs fed with high soy hull fibre. The results of this present study imply that the addition of POM may improve the total microbial composition hence increase the digestibility of crude fibre. The mechanism through by a low concentration of POM increased nutrient digestibility is unclear. However, it was well established that mushrooms contain high fibre content^9^ and these dietary fibres may decrease intestinal transit time and hence account for its effect on nutrient digestibility.^19^, ^38^

### Effects of dietary POM levels on fecal pH and SCFAs

Mushrooms contain bioactive compounds and nutrients such as high fibre.^9^, ^39^ These high fibre levels are less digestible in the small intestine as a result of the immature gastrointestinal tract of piglets and the absence of fibre digesting enzymes. However, microbes in the hindgut can ferment these fibres to synthesize SCFAs, such as acetate, butyrate and propionate. These SCFAs account for approximately 90% of the total acid production in the hindgut of monogastric animals, therefore representing approximately 10–30% of the total energy requirement of animals.^40^ The results of the present study were consistent with the findings by Bikker *et al.* 41 who reported that feeding high fermented carbohydrate diets to newly weaned piglets increased the total volatile fatty acids, acetic acid and butyric acid levels. Che *et al.* 27 reported an increase in the acetic acid, butyric acid, propionic acid and total volatile fatty acid levels after supplementing 2.50% *A. membranaceus* fibre to the diet of piglets. By contrast, Freire *et al.* 19 found that the total volatile fatty acids level in the cecum of weaning piglets decreased at a ratio of 1.16 mg g^−1^ after changing the diet from sugar beet pulp to soybean hulls. Furthermore, Piva *et al*_._ 42 showed that the pH of the colon might affect microbial fermentation and volatile fatty acids production rather than influencing dietary acid synthesis. Similarly, there was no effect on the fecal and ilea pH level in early weaned piglets after dietary supplementation of herbal extracts.^43^

### Effects of dietary POM levels on serum cytokines and immunoglobulins

Mushrooms contain secondary metabolites such as acids, alkaloids, lactones, polyphenols, sesquiterpenes, sterols, terpenoids, polysaccharides (*β*‐glucans) and glycoproteins.^44^ These *β*‐glucans are responsible for the anticancer, immunomodulation, anticholesterolemic, antioxidant and neuroprotective effects of most edible mushrooms.^44^ Oyster mushrooms are used in traditional Chinese medicines to stimulate both innate and adaptive immunity.^11^ The results of the present study were in agreement with the findings by Li *et al.* 45 who reported an increase in serum IgG and IL‐2 levels in pigs treated with *Ganoderma lucidum* extracts. Similarly, Lv *et al.* 46 reported that dietary supplementation with glutamine increased the concentration of IgA, IgG, or IgM in weaned piglets. Xi *et al.* 47 also reported a significant increase in the IL‐2 and IFN‐γ levels in chicken after feeding on the ultrafine powder extracted from the stem and leaves of *Astragalus*. In addition, Feng *et al.* 48 showed that feeding *Aspergillus oryzae* fermented soybean meal increased (*P* < 0.05) the serum IgM concentration in broilers throughout the entire trial period, although the IgA level only increased in the peak growth period.

### Effects of dietary POM levels on fecal microbial composition

The variations in microbial count within the intestines can have a constructive effect on the colonization and the activities of the intestinal microorganisms, digestion, absorption, biotransformation and consequently affect productivity.^36^ Synytsya *et al.* 49 indicated that extracts of *P. ostreatus* and *Pleurotus eryngii* increased the growth of intestinal microbes such as *Lactobacillus* species, *Bifidobacterium* species and *Enterococcus faecium*. In the present study, the OTU analysis represents the fecal microbial diversity in all of the treatment groups. The alpha diversity indices represent the species richness and evenness of individual samples. Furthermore, the taxonomic classification of the microbial composition in the fecal samples of piglets revealed that the *Bacteroidetes*, *Firmicutes*, *Cyanobacteria*, *Proteobacteria, Spirochaetes*, *Chloroflexi* and *Actinobacteria* were the dominant bacterial phyla. These were consistent with the findings by Che *et al.* 27 who reported that the *Bacteroidetes, Firmicutes, Proteobacteria, Spirochaetes*, *Cyanobacteria* and *Chloroflexi* were the dominant bacterial phyla in the cecum of piglets fed *A. membranaceus* fibre. In addition, Lamendella *et al.* 50 reported that the *Firmicutes* and *Bacteroidetes* were the dominant phyla in pig fecal microbiota.

The results of the present study showed a higher population of *Bacteroidetes* in the 5.00 g kg^−1^ POM and the control group compared to the other treatment groups. This may be a result of individual animal variation. The relative abundance of the *Firmicutes* was higher in the 10.00 g kg^−1^ POM treated group compared to the other treatments. Macfarlane *et al.* 51 reported that the composition of the microbiota could be influenced by several factors, such as age, antibiotics, diet, disease, drugs and other factors. The findings of the present study were in agreement with the results of Pajarillo *et al.* 52 who reported that the *Firmicutes* and *Bacteroidetes* were the dominant (90%) phyla in the fecal bacterial community at the time of weaning. Yin *et al.* 53 reported that the two main phyla constituting the gut microbiota of mice were the *Firmicutes* and *Bacteroidetes* after supplementing high‐fat and melatonin to the diet of mice. By contrast, Yin *et al.* 54 reported that the *Firmicutes* and the *Proteobacteria* were the two main bacterial phyla in the gut microbial of piglets during dietary lysine restriction. Tannock^55^ reported that the physiological and dietary factors were the key conditions that may influence the composition and abundance of the intestinal microbiota. Regardless of the dietary treatment in the present study, the microbial composition of each individual piglet was unique (Fig. 6). Previous studies supported the findings in which pigs fed with different diets exhibited unique microbial population in their feces, as analyzed by Illumina‐based sequencing and pyrosequencing of the 16S rDNA library technology.^52^, ^56^, ^57^ At the family level, the results of the present study revealed that the *Prevotellaceae*, *Veillonellaceae*, [*Paraprevotellaceae*], *Lachnospiraceae*, *Ruminococcaceae*, *Succinivibrionaceae* and S24‐7 were the dominant families in all of the samples. Similarly, Che *et al.* 27 reported that the *Prevotellaceae, Veillonellaceae, Paraprevotellaceae, Lachnospiraceae, Ruminococcaceae* and *Succinivibrionaceae* were the dominant families in the cecum samples after supplementing *A. membranaceus* fibre to the diet of piglets. The relative abundance of the *Prevotellaceae* was higher in the 5.00 g kg^−1^ POM treated group and the control group. However, the relative abundance of the *Veillonellaceae* was higher in the 10.00 g kg^−1^ POM treated group. Comparatively, the population of the *Veillonellaceae* was relatively low in all of the treated groups compared to the *Prevotellaceae*. Recent studies by Kim *et al.* 57 and Daly *et al.* 58 revealed a significantly lower abundance of the *Veillonellaceae* in the fecal microbes of pigs raised on a commercial feed. In addition, there was a decrease in the relative abundance of the *Ruminococcaceae* in the POM supplemented groups compared to the control group. The reduction in the relative abundance of the *Ruminococcaceae* in the POM treated groups may be a result of the increase in the SCFAs levels in the pig cecum.^59^ At the genus level, the results of the present study revealed that the *Prevotella*, *Anaerovibrio*, *Phascolarctobacterium*, [*Prevotella*], *Megasphaera*, *Succinivibrio*, *Roseburia*, *Ruminococcus* and *Oscillospira* were the dominant genera in all samples. There was a significant reduction in the relative abundance of *Anaerovibrio* genera in the 15.00 g kg^−1^ POM treated group compared to the other treatment groups. However, there was a significant increase in the *Megasphaera* genera in the 10.00 g kg^−1^ POM treated group compared to the other treatments. The reasons for this increase in the relative abundance of *Megasphaera* in the 10.00 g kg^−1^ POM and a decrease in the *Anaerovibrio* genera in the 15.00 g kg^−1^ POM treated groups were unclear.

In conclusion, the present study provides some valuable insights with respect to the replacement of antibiotics in monogastric feeding and management. Dietary supplementation with POM might increase growth performance and SCFAs production, which might result in a decrease in diarrhea occurrence and, consequently, increased intestinal microbial population and diversity. The present study found that the dietary inclusion of 5.00–15.00 g kg^−1^ POM might increase the overall performance of piglets.
